# Environmental influences on neural systems of relational complexity

**DOI:** 10.3389/fpsyg.2013.00631

**Published:** 2013-09-26

**Authors:** M. Layne Kalbfleisch, Megan T. deBettencourt, Rebecca Kopperman, Meredith Banasiak, Joshua M. Roberts, Maryam Halavi

**Affiliations:** ^1^KIDLAB, Krasnow Institute for Advanced Study, George Mason UniversityFairfax, VA, USA; ^2^Graduate Neuroscience, College of Science, George Mason UniversityFairfax, VA, USA; ^3^College of Education and Human Development, George Mason UniversityFairfax, VA, USA; ^4^Department of Pediatrics, The George Washington School of Medicine and Health SciencesWashington, DC, USA; ^5^Laboratory for Intelligent Imaging and Neural Computing, The Fu Foundation School of Engineering and Applied Science, Columbia UniversityNew York, NY, USA; ^6^College of Architecture and Planning, University of ColoradoBoulder, CO, USA; ^7^Cognitive and Behavioral Neuroscience Program, Department of Psychology, College of Humanities and Social Sciences, George Mason UniversityFairfax, VA, USA

**Keywords:** event-related fMRI, heuristic processing, prefrontal cortex, reasoning, color perception, relational complexity, visual contrast, constructivist learning

## Abstract

Constructivist learning theory contends that we construct knowledge by experience and that environmental context influences learning. To explore this principle, we examined the cognitive process relational complexity (RC), defined as the number of visual dimensions considered during problem solving on a matrix reasoning task and a well-documented measure of mature reasoning capacity. We sought to determine how the visual environment influences RC by examining the influence of color and visual contrast on RC in a neuroimaging task. To specify the contributions of sensory demand and relational integration to reasoning, our participants performed a non-verbal matrix task comprised of color, no-color line, or black-white visual contrast conditions parametrically varied by complexity (relations 0, 1, 2). The use of matrix reasoning is ecologically valid for its psychometric relevance and for its potential to link the processing of psychophysically specific visual properties with various levels of RC during reasoning. The role of these elements is important because matrix tests assess intellectual aptitude based on these seemingly context-less exercises. This experiment is a first step toward examining the psychophysical underpinnings of performance on these types of problems. The importance of this is increased in light of recent evidence that intelligence can be linked to visual discrimination. We submit three main findings. First, color and black-white visual contrast (BWVC) add demand at a basic sensory level, but contributions from color and from BWVC are dissociable in cortex such that color engages a “reasoning heuristic” and BWVC engages a “sensory heuristic.” Second, color supports contextual sense-making by boosting salience resulting in faster problem solving. Lastly, when visual complexity reaches 2-relations, color and visual contrast relinquish salience to other dimensions of problem solving.

## Introduction

The human brain collates, integrates, and binds basic sensory inputs that result in our capacities to think, reason, and perform at high levels (Waltz et al., 1999; Kalbfleisch et al., 2006, 2007). It processes with great efficiency in complex environments such as natural scenes, virtual worlds, gaming environments, athletics, the visual and performing arts, and in the classroom, to name a few. Studying learning and performance in those contexts means accepting a high degree of variance in the assessment and evaluation process. Contemporary learning theory (e.g., constructivism) highlights the central role of both the learner's meaning making and his or her interaction with the environment in creating knowledge. Constructivism is a learning theory that contends we construct knowledge best by experience and that our environment and the context it provides is a central determinant in the quality of our learning. It holds within it such principles as “scaffolding,” “the zone of proximal development,” and the “genetic epistemology” that have influenced the fields of educational psychology and the learning sciences for decades (Vygotsky, 1928; Bruner, 1960, 1990; Piaget, 1970). Educational psychology has held a long interest in determining more specific heuristics that would increase our pragmatic capacities to implement constructivist principles of learning (Phillips, 2006). In past, neuroscience has examined the means by which experience impacts gray matter change (Diamond et al., 1975, 1976; Quartz and Sejnowski, 1997), however, there is a paucity of evidence for the consequences of specific environmental influence on functional neuroplasticity during higher-level reasoning. A better understanding of how the brain achieves efficiency with input from the physical environment will contribute insight to help education researchers, designers of architecture, technology, and curriculum, and teaching practitioners gain a more practical understanding of constructivist principles so that they can accommodate and apply them in practice with greater precision.

Early experiments exploring aspects of visual complexity such as pattern, shape, color and contour determined that features of symmetry and context reduce the psychological perception of complexity (Chipman, 1977). Relating to this, we aim to understand the neural basis of some of these processes during problem solving and how or why they may support constructivist learning. The key question is how do we identify and trace neural systems of reasoning that are engaged during psychometric tests and controlled in neuroimaging paradigms to better appreciate how these systems evolve and support more naturalistic knowledge about cognition (Pylyshyn, 1999; Mahon and Caramazza, 2009; Schwarzkopf et al., 2009)? Specifically relating to our experimental design and the opportunity to connect a neural result with a meaningful outcome for education and measurement, we aim to understand more about how and/or why certain visual properties or heuristics are enlisted during matrix reasoning because it serves as a proxy measure of intellectual capacity (Kalbfleisch, 2004). We employ the word “heuristic” to represent how visual and reasoning systems function together to compute “empirical approximations” (Gershman et al., 2012) or create “frames” that assist in simplifying context during ambiguous or complex reasoning conditions (De Martino et al., 2009). De Martino et al. (2009) highlight how affective heuristics influence rational decision-making. In a similar fashion, to examine how qualities of visual perception influence relational complexity (RC), we apply black-white visual contrast (BWVC) and color to an ecologically-valid model of non-verbal matrix reasoning. Our model is ecologically valid in two ways, one, for its psychometric relevance, and two, for its potential to link the processing of psychophysically specific visual properties with various levels of RC during reasoning. The role of these fundamental perceptual psychophysical elements is important because non-verbal psychometric instruments such as matrix reasoning tests assess one's aptitude from how well-one problem solves within these seemingly context-less exercises. In doing this, we stand to gain a better understanding of how specific stimulus properties converge to influence general neural systems affiliated with human reasoning (Christoff and Owen, 2006). This experiment is a first step toward examining the psychophysical underpinnings of performance on these types of problems.

Recent letters postulate on how to reconcile results among neuroimaging studies of reasoning (Brzezicka et al., 2011) and the potential application of this knowledge for training, pedagogy, and intervention (Houde, 2008). It stands, then, that matrix reasoning (a measure of intellectual capacity that we can parametrically vary in an experimental context) correlates with some forms of academic achievement (evidence of behavior in an important real-world setting, the classroom), and presents a ripe opportunity to explore how specific visual properties from complex real-world environments, may impact performance potential across a range of individual differences. Underscoring the relevancy of this idea, emerging evidence asserts there may be a predictive validity in the relationship between intelligence and the sensory discrimination of visual motion (Melnick et al., 2013).

In keeping with this, the evolutionary importance of visual contrast on perception (Kelly, 1977), illustrates that some aspects of early visual processing such as color and visual contrast are important and central to survival (Bowmaker, 1998; Gerl and Morris, 2008). Studies to date that examine how the visual system is modulated by specific properties such as color or motion are often explored within experiments on perception that have no overt problem solving requirement (Shipp et al., 2009; Cardin et al., 2011). Here, we examine these properties within the construct typically characterized in the superior frontal lobes called “RC,” “the number of related dimensions, or sources of variation, that need to be considered simultaneously in order to arrive at a correct answer” (Halford and Wilson, 1980). RC is both a perceptual process and a metric of visual complexity that supports mature reasoning capacity (Halford et al., 1998; Christoff et al., 2001; Kroger et al., 2002; Crone et al., 2009; Wendelken and Bunge, 2010). It is a systematic heuristic that accounts for how visual properties scale in complexity and, for this reason, provides an ideal method for exploring the means by which visual properties from the environment such as color and visual contrast influence higher-level reasoning and choice. In general, relational integration relies only in part on the executive resources of working memory (Cho et al., 2007; Badre, 2008) and attention (Posner and Petersen, 1990). Previous experiments have shown that reasoning involves, but is separate from working memory (McCarthy et al., 1994; Owen et al., 1996; Bechara et al., 1998; Ruff et al., 2003; Cowan et al., 2012). In fact, functional nuclei are distributed throughout the human frontal lobes that enable diverse executive functions (Duncan and Owen, 2000), though they appear uniform to the human eye. In this paper, we give central focus to RC, leveraging its parametric properties to scaffold between and among the visual properties color and BWVC during matrix reasoning.

Color as a visual feature in both low-level and high-level vision has been well-corroborated by converging neurophysiological, neuropsychological, and behavioral evidence (Livingstone and Hubel, 1987; Bartels and Zeki, 2000; Tanaka et al., 2001; Wade et al., 2002; Shipp et al., 2009; Zeki and Stutters, 2013). Color facilitates object perception and also recognition, but has also been shown to play a significant role in scene segmentation and visual memory (Tanaka et al., 2001; Gegenfurtner, 2003; Werner and Chalupa, 2003; Peelen et al., 2009). Brain areas necessary for color perception and integration include several areas in the visual cortex such as ventral occipital and temporal extrastriate areas (Wade et al., 2008), and sub-regions of the occipital and parietal lobe (Vaina, 1994; Wade et al., 2002) in fusiform gyrus, collateral sulcus, and lingual gyrus (Bartels and Zeki, 2000; Claeys et al., 2004; Morita et al., 2004). Thus, it is important to determine when color serves basic perception and when it is involved in higher level processing. Important to this experiment, selecting color on its own in a choice task does not activate the superior or middle frontal cortices (Rowe et al., 2005). Whereas, RC is supported by activity from the superior and lateral areas of the prefrontal cortex, known collectively as the rostrolateral prefrontal cortex or RLPFC (Christoff et al., 2001; Kroger et al., 2002; Koechlin and Summerfield, 2007; Bunge et al., 2009).

To summarize, color perception and RC are separately accounted for in the neuroimaging literature, but how the brain integrates properties of visual information from the environment such as color and visual contrast into the RC process and higher-level reasoning in general is unknown.

**Figure 1:**
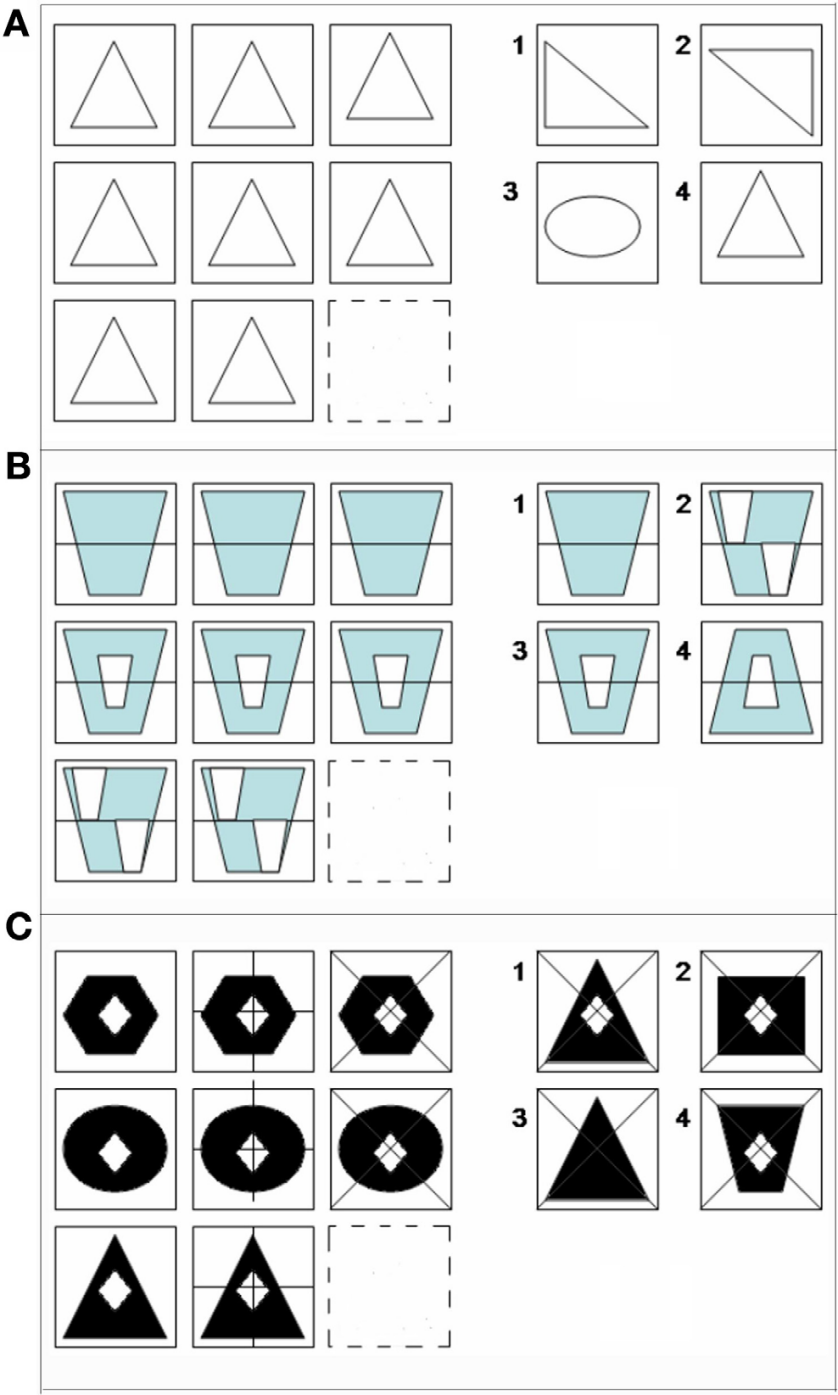
**Examples of matrix reasoning stimuli at (A) 0-relational, (B) 1-relational, and (C) 2-relational complexity.** All matrix problems were experienced in No-Color **(A)**, Color **(B)**, and Black-white visual contrast **(C)** conditions.

On a practical level, several cognitive neuroscience studies have based neuroimaging tasks on problems adapted from the Raven's Progressive Matrices Test (RPM; Raven, 1938) to document reasoning and RC during fMRI, functional magnetic resonance imaging (Prabhakaran et al., 1997; Christoff et al., 2001; Kroger et al., 2002; Crone et al., 2009; Baldo et al., 2010). The RPM, a psychometric assessment of intellectual capacity, is designed to evaluate the ability to form perceptual relations and to reason by analogy independent of language, ethnicity, disability, or formal schooling. As such, it offers a way to examine consilient real-world visual perception and its influence on decision-making. Here, it is a logical extension to use an RPM-like matrix task to parametrically examine the impact of color and visual contrast on RC. This self-paced fMRI study employs RPM-like stimuli designed to parametrically vary complexity (0, 1, 2) within no-color line, BWVC and color conditions (Figure 1).

Parametrically varying visual properties at the same level of reasoning complexity provides us with a strategy to document the sensory contributions to reasoning between each kind of property. In turn, parametrically varying the complexity level within each visual property permits us to characterize the neural systems supporting reasoning among cognitive levels of demand within each type of stimulus context. We hypothesized that color and visual contrast (1) contribute additional sensory load to relational reasoning resulting in extended behavioral reaction times, and (2) that those differences are observable and dissociable in the visual and prefrontal cortices. Thus, hypothesizing that real-world visual properties play a supportive role in relational reasoning, our open question was under which levels of complexity and to what extent do properties of the visual environment influence the RC system in the frontal cortex to enable successful decision making and choice?

To our knowledge, this study is the first to document the convergence of color perception and RC during a self-paced reasoning task, representing one example of how perceptual processes enable higher level cognition in the context of a task format commonly used to assess problem solving skill and general intellectual capacity.

## Materials and methods

### Subjects

Participants included 34 neurologically healthy volunteers (11 male, 23 female; mean age = 24.2 years, range 18–46 years, *SD* = 7.5). All volunteers were right-handed with a mean score of 86.48% on the Edinburgh Handedness Inventory (Oldfield, 1971). None reported history of color-blindness. All participants gave written informed consent prior to participating in the experiment approved by the Human Subjects Research Board (HSRB) of George Mason University, Fairfax, VA. Each subject's participation consisted of one visit to the laboratory and involved three parts: a confirmatory MRI safety and compliance screening by the technologist, a brief practice session with the task using problem trials separate from the problems they solved in the scanner, and ~1 h in the MRI scanner including time for the subject to be comfortably positioned in the scanner, to acquire structural brain scans needed for data analysis, and to have the participant perform three runs of the functional task described below.

### Task design

The color relational complexity task (CRC) is a self-paced, event-related design incorporating 3 × 3 matrix reasoning problems missing the bottom right figure in No-color line (NC) (Figure 1A), Color (Figure 1B), and BWVC conditions (Figure 1C). The event-related design prevents an attention confound as the participant cannot anticipate when the next trial will appear. The task is self-paced allowing the participant to solve each problem at their own speed, but the presentation of new trials following the one just solved were jittered at intervals based on the 3s repetition time in the scanning protocol for fMRI (3, 6, 9, 12, 15 s). This jitter controls for any carryover effect of the blood oxygen level dependent (BOLD) signal, securing the opportunity to timelock the specific BOLD response to the particular time point when the subject solved each problem trial. Three levels of complexity (0, 1, and 2) are defined according to the original definition as the number of variations in a visual matrix stimulus that are simultaneously processed during reasoning (Halford and Wilson, 1980). To solve, participants consider the relationships among 8 of 9 provided pieces of the matrix in the trial and select the correct answer from four choices provided. To prohibit learning and fatigue confounds, the experiment consisted of three runs presented in counterbalanced order. Each run, ~14.5 min in length, contained 13 trials at 0-, 1-, and 2-relational complexity for Color, NC, and BWVC for a total of 9 conditions presented in random order (randomized in an Excel-based program to optimize randomization). Zero-relational problems contained no change in relationship between rows or within columns of the matrix and no relational processing was necessary to solve (Figure 1A). One-relational trials are defined by a single change between rows of the matrix (Figure 1B). Two-relational trials are defined by two changes, both between columns and within rows of the matrix (Figure 1C). Items were either drawn in no-color line (NC), filled with black and white (BWVC), or filled with light blue (Color), a color universally perceived by individuals with typical color perception as well as those with most common forms of color blindness (Abramov and Gordon, 1994; Deeb, 2004). NC conditions provided a baseline control and validation of previous studies of RC using these item types. BWVC conditions provided a control for Color conditions, permitting us to examine the unique contributions to sensory and/or cognitive processing from each visual property.

Prior to their functional magnetic resonance imaging (fMRI) session, participants performed a practice test with samples of trials at different complexity levels to avoid a learning confound. In the MRI, they had a brief practice with the button box, using their right hand index, middle, ring, and small fingers to press one of four buttons corresponding to their choice. Problem trials were presented from a computer and back-projected through a wave-guide onto a screen located in the back of the MRI scanner bore. Visual images were viewed from a mirror mounted on the head coil above the participant's head.

**Figure 2:**
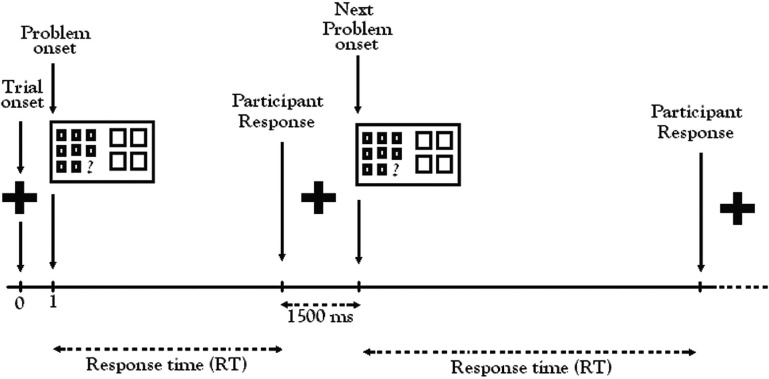
**Color relational complexity (CRC) fMRI task sequence.** CRC task was a self-paced, event-related design with runs administered in counterbalanced order. Color, No-Color, and Black-white visual contrast matrix items were randomly distributed across three functional runs containing 13 trials of each condition at 0, 1, and 2 levels of complexity.

During the fMRI experiment, participants were alerted to the beginning of each trial by a visual cue (a 1500 ms fixation cross, “+”) followed by a matrix problem (Figure 2). Working at their own pace, participants indicated their choice by pressing one of the four buttons on the button-box in their right hand. For each trial, the time from start of the problem to the response defined the response time (RT). The random order of trials and conditions in each run created sufficient variability in the RT to prevent autocorrelation of BOLD responses, the basis of the fMRI signal. After each response, participants were presented with the visual fixation cue (“+”) to rest before the next trial. Participants were instructed to concentrate more on accuracy than speed.

### MRI data acquisition

Structural and functional MRI data were acquired using a 3.0-tesla Siemens Allegra head-only scanner (Siemens Medical Solutions, USA) located at the Krasnow Institute for Advanced Study at George Mason University. A CP TX/R head coil single-channel was used during data collection and head motion was restricted with memory foam inserts to secure and cushion the head within the head coil. Functional runs were acquired using a standard BOLD (blood-oxygenation-level-dependent) gradient-echo echo-planner imaging (EPI) pulse sequence (*TR* = 3000 ms, *TE* = 30 ms, flip angle = 70°, *FOV* = 192 × 192 mm^2^, 64 × 64 voxels). Functional images covered the whole brain, collected in 870 volumes per participant over the course of 3 runs of 14.5 min each. Each volume consisted of 48 interleaved slices with 0.2 mm gap and thickness of 3.0 mm. The first three volumes were discarded in order to account for signal stabilization. The start of each run was manually synchronized with the scanner.

Structural images were collected as high-resolution T1-weighted images using a MPRAGE (magnetization-prepared rapid-acquisition gradient-echo) sequence with following parameters: (*TR* = 2300 ms, *TE* = 2.7 ms, flip angle = 12°, matrix size of 256 × 256) and additional structural images with following parameters: spin-echo axial-oblique T1-weighted structural scans of the whole brain (coplanar with functional images, 48 slices, repetition time (*TR*) = 200 ms, echo time (*TE*) = 3.6 ms, field of view (*FOV*) = 192 × 192 mm^2^; slice thickness = 3 mm, flip angle = 75°; matrix size = 205 × 256).

### Preprocessing

Image reconstruction was performed offline. The conversion of raw data completed using MRIconvert (University of Oregon Lewis Center for Neuroimaging). Data processing and analysis were carried out using the Statistical Parametric Mapping software package SPM5 (Wellcome Trust Centre for Neuroimaging, London, UK). Pre-processing included slice-timing, reorientation, and realignment using INRIAlign (Freire and Mangin, 2001; Freire et al., 2002), normalization to SPM EPI template, and spatial smoothing with 9 mm full width at half maximum (FWHM) isotropic Gaussian kernel. Motion inclusion criteria (less than 2 mm translational and less than 2° rotational movement) were met for all runs incorporated into the analysis. A high-pass filter with a cut-off period of 128 s was used to remove low-frequency drifts unrelated to the experimental paradigm.

### fMRI analysis

A general linear model (GLM) was applied to the time course of activations to estimate condition effects at each voxel. In the first-level single subject analysis, the response function was modeled as an RT-based boxcar function (as described in the methods of Christoff et al., 2001) matching the onsets of the stimulus presentation time convolved with a canonical hemodynamic response function (HRF) (Friston et al., 1994). Linear contrast of estimated regression coefficients were used to compare the specific effects of each condition. The fMRI data from each participant were used to generate statistical contrasts for brain activations. Single runs of single subjects were evaluated to disqualify for activations outside of neural tissue and artifacts. The statistical parametric maps from the *t*-statistic of each voxel value for each contrast in first level analysis were entered into second-level group analysis. A random effects model was used to account for both scan-to-scan and subject-to-subject variability. Relevant to this experiment, random effects models assume that measured effects vary across the population and can account for inter-subject variance in the statistical analysis. This preserves opportunity to draw inferences at the group level. In the second-level analysis, one-sample *t*-tests were applied to the first-level statistical parametric maps for correlation analyses of each parameter with the BOLD signal corrected for multiple comparisons using false discovery rate (FDR,*p* < 0.05).

FDR is a type of Bonferroni correction suited to fMRI data analysis. A classic Bonferroni correction algorithm is too severe to apply to the multiple comparisons problem in fMRI data because of the comparison of over 30,000 individual voxels that comprise a single subject brain map. The consequences of this correction decreases Type I error rate, producing fewer false positives or increases Type II error rate. Additionally, it is not appropriate for correlated data and most fMRI data has significant correlation due to the fact that an individual's brain map is parcellated into these thousands of voxels and then binned into a larger group analysis that compounds these comparisons. Specifically, FDR controls the expected proportion of false positive values based on the observed distribution of activity making it more sensitive than the classic Bonferroni correction (Genovese et al., 2002). Here, statistical maps were generated based on the FDR correction and cluster extent sizes of more than 7 voxels identified the neural areas significant for each contrast.

### ROI analysis

Region of interest (ROI) analysis was used to address our a priori hypothesis that the RLPFC would support RC within a stimulus context that included real world visual properties. To explore the signal more precisely for Color 1- and 2-relational problems (the levels of complexity robust enough to initiate neural systems that support RC), we performed both structural and functional ROI analysis. Structural ROIs were created according areas identified from previous studies that used similar paradigms (Christoff et al., 2001; Kroger et al., 2002). Analysis was done using MarsBar, version 0.41 (Brett et al., 2002). ROIs were defined using the WFU Pickatlas, the AAL, and Talairach Daemon atlases (Lancaster et al., 1997; Tzourio-Mazoyer et al., 2002; Maldjian et al., 2003, 2004). For each ROI, the MNI coordinates of the maximums of signal intensity for each activation cluster were entered into the WFU Pickatlas to determine anatomical location. All voxel classifications were confirmed after translation from MNI into Talairach coordinates by manual visual inspection using the Talairach atlas (Talairach and Tournoux, 1988). Functional ROI analysis was performed in the RLFPC (BA6, 8, 10) and posterior areas surviving correction for multiple comparisons by creating a ROI from each maxima in the cluster analysis and masking it with spherical ROIs (radius = 10 mm) at the peaks of activation in each region cluster (Poldrack, 2007). These ROIs were analyzed statistically on a single subject and group level for statistical power for comparisons and for the finite impulse response (FIR) time course of the activations in Color 1- and 2-relational problems.

## Results

As a précis to reporting these results, we direct the reader's attention to a few primers that promote greater scientific understanding of neuroscience tools and methods in education and social science research (O'Boyle and Gill, 1998; Cacioppo et al., 2003; Kalbfleisch, 2008). Publications such as these educate the layreader about these tools and methods and lay plain the potential impact of neuroimaging studies to improve how we educate. In addition, to present an advanced organizer to the reader, what do the results from this experiment suggest in practical terms?

First, our main behavioral findings can be understood in two points:

1. As expected, RTs increase as a function of complexity within each condition.
2. Counter-intuitive to a parametric response to increased cognitive load, RT during Color 1-relation was significantly different from and less when compared with NC 1-relation and with BWVC 1-relation illustrating that color facilitates cognitive performance by boosting salience.

Second, our main physiological findings can be understood in two points:

1. When controlling for sensory contribution, a broader, larger, and functionally distinct suite of visual areas support processing in BWVC (BA17/18/19) than for Color (BA18/19), illustrating a neural efficiency for how color primes cognitive performance.
2. When controlling for stimulus complexity, the rostrolateral prefrontal cortex (RLPFC), the suite of areas that specifically support relational integration during reasoning, is preferentially engaged during Color RC. This is observed in a double dissociation between visual and frontal areas that engage for BWVC (working memory) and for Color (RC) (Table 3, Figure 4).

### Behavioral results

**Figure 3:**
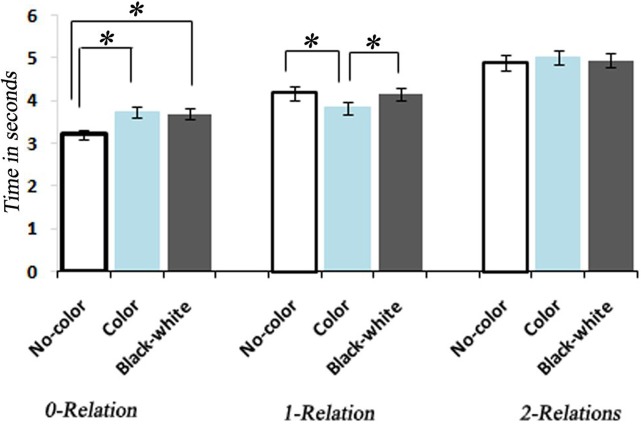
**Bar graph representation of response times associated with No-Color, Color, and Black-white Visual Contrast task itemsfor 0, 1, and 2 levels of relational complexity.** *, indicates a statistically significant difference between conditions.

Participants demonstrated a mean level accuracy of 95.7% (standard error = 0.44%). The analysis of data for systematic decrease in accuracy or systematic increase in RT from the beginning to the end of each run did not indicate a fatigue effect. Accuracy percentage and mean RT per condition were computed for each participant and averaged for each condition and complexity level (Figure 3). As the task was self-paced, observed changes in accuracy between conditions were not significant. Only correct trials were included in the RT analysis. Mean RTs for each condition at each level of complexity are reported in Table 1.

**Table 1:** **Descriptive statistics for category specific performance—Black-White Visual Contrast (BWVC), Color (C), and No-Color (NC) at 0, 1, and 2 levels of Relational Complexity**.

**Contrast**	**Complexity level**	**Mean reaction time (ms)**
BWVC	2	4870
	1	4032
	0	3636
Color	2	5062
	1	3780
	0	3743
No-color	2	4991
	1	3984
	0	3138

A One-Way ANOVA with repeated measures and a *post-hoc* analysis of RTs showed a significant increase corresponding to the increase in the level of complexity within each condition [*F*_(8, 34)_ = 129.93, *P* < 0.0001] (Figure 3). A between comparison of both Color and BWVC with No-Color problems at equal complexity levels showed significant increase in RT for BWVC 0-relational trails (*M* = 475.99 ± 61.34 SE,*P* < 0.000) and for Color 0-relational trails (*M* = 525.58 ± 66.99 SE,*P* < 0.000). However, the same comparison at 1-RC level showed a significant decrease in RT for Color problems (*M* = 234.60 ± 66.02 SE,*P* < 0.04) and no significant change for BWVC. Within 2-RC level, no significant changes were assessed between No-Color and Color or between No-Color and BWVC. A comparison between Color 0-relation and BWVC 0-relation trials did not show any significant change.

### fMRI results

Neuroimaging results include 34 participants and report activations identified through voxel-based analysis using an RT-convolved HRF. We analyzed the results for the effect of complexity level (0, 1, 2) and for the effect of each condition (NC, BWVC, and Color). For complexity level, contrasts were analyzed within and between conditions by evaluating 1-relational vs. 0-RC and 2-relational vs. 1-RC. For the effect of each condition, contrasts were performed between conditions at equal level of complexity. All neuroanatomical results are reported in Tables 2, 3. To facilitate the reading of the prose, neuroanatomical areas are referred to in the convention of Brodmann Areas, a standard “shorthand” for associating neuroanatomy with function (http://en.wikipedia.org/wiki/Brodmann_area). Brodmann Areas are then listed in the Tables 2–4 to permit a more specific report of significantly correlated areas in cortex characterized in this experiment. Results presented here survive a statistical correction for multiple comparisons at a false discovery rate (FDR) threshold *p* < 0.05.

**Table 2:** **Sensory contributions to reasoning—neuroanatomical results for random-effects analysis between BWVC, Color, and NC conditions at 0, 1, 2 complexity**.

**Regions of activations**	**BA**	**Cluster voxel size**	**MNI coordinates**			***Z*-score**
**Anatomical label**		**(uncorrected value)**		
			***x***	***y***	***z***	
**BLACK-WHITE 2-RELATION > NO-COLOR 2-RELATION**						
**Occipital**						
L Lingual gyrus	18	375	−9	−82	2	4.94^*^
L Lingual gyrus	18		−24	−76	−9	4.64^*^
R Lingual gyrus	19		24	−59	−5	4.49^*^
**BLACK-WHITE 1-RELATION > NO-COLOR 1-RELATION**						
**Occipital**						
L Cuneus	17	248	−9	−90	7	5.58^*^
L Lingual gyrus	18		−6	−76	4	5.49^*^
R Lingual gyrus	19		21	−67	−4	5.10^*^
L Lingual gyrus	18	50	−18	−70	−7	4.30^*^
L Fusiform gyrus	19		−21	−62	−10	4.25^*^
L Lingual gyrus	18		−33	−74	−9	3.62^*^
**BLACK-WHITE 0-RELATION > NO-COLOR 0-RELATION**						
**Occipital**						
L Lingual gyrus	19	141	−30	−64	−5	5.26^*^
L Middle occipital gyrus	19	72	−48	−78	9	4.94^*^
R Lingual gyrus	19	129	21	−70	−4	7.89^*^
R Fusiform gyrus	19		30	−62	−7	4.72^*^
L Cuneus	18	34	−9	−87	13	4.09^*^
R Superior occipital gyrus	19	42	33	−80	23	4.05^*^
**Temporal**						
R Middle temporal gyrus	19		45	−78	20	3.92^*^
**COLOR 2-RELATION > NO-COLOR 2-RELATION**						
**Occipital**						
L Lingual gyrus	18	47 (147)	−27	−78	−15	4.69^*^
	18		−12	−84	−9	4.34^*^
R Fusiform gyrus	19	(25)	30	−69	−18	3.88
L Fusiform gyrus	19	(17)	−27	−51	−18	3.63
**COLOR 1-RELATION > NO-COLOR 1-RELATION**						
**Occipital**						
R Lingual gyrus	18	297 (324)	27	−57	−12	5.98^*^
	18		30	−69	−12	5.81^*^
L Lingual gyrus	18	318 (355)	−30	−60	−15	5.30^*^
L Cuneus	18	39 (49)	−9	−96	18	4.69^*^
R Cuneus	18	89 (99)	15	−99	21	4.32^*^
**COLOR 0-RELATION > NO-COLOR 0-RELATION**						
**Frontal**						
L Precentral gyrus	4	23	−33	−18	39	4.02
L Middle frontal gyrus	10	10	−36	45	9	358
**Cerebellum**						
L Posterior lobe		15	−6	−40	−40	3.94

All regions consist of atleast 7 voxels with an uncorrected P < 0.001;

* significance P < 0.05 False Discovery Rate (FDR) correction.

**Table 3:** **Influence of visual properties on neural systems of relational complexity—neuroanatomical results for the random-effects analysis within Color and BWVC conditions**.

**Regions of activations Anatomical label**	**BA**	**Cluster voxel size (uncorrected value)**	**MNI coordinates**			***Z*-score**
			***x***	***y***	***z***	
**COLOR 2-RELATION > COLOR 1-RELATION**						
**Frontal**						
L Middle frontal gyrus	6	38 (53)	−30	6	60	5.03^*^
R Medial frontal gyrus	8	33 (53)	6	18	48	4.88^*^
R Middle frontal gyrus	10	20 (57)	45	54	−3	4.84^*^
L Middle frontal gyrus	10	(7)	−36	57	18	3.54
**Parietal**						
L Superior parietal lobule	7	72 (195)	−33	−63	57	4.78^*^
R Precuneus	7		21	−78	51	3.88^*^
R Superior parietal lobule	7	38 (90)	33	−63	54	4.19^*^
L Precuneus	7		−3	−66	54	4.17^*^
**Occipital**						
R Inferior occipital gyrus	18	29 (44)	30	−99	−3	5.00^*^
L Lingual gyrus	18	(19)	−24	−102	−3	3.97
**BLACK-WHITE VISUAL CONTRAST 2-RELATION > BLACK-WHITE VISUAL CONTRAST 1-RELATION**						
**Frontal**						
R Middle frontal gyrus	9	(21)	39	13	27	3.81
**Parietal**						
L Postcentral gyrus	4	(19)	−39	−21	45	4.11
L Postcentral gyrus	2		−45	−27	37	3.75
L Postcentral gyrus	2		−33	−27	40	3.48
R Inferior parietal lobule	40	(10)	33	−53	41	3.56
**Occipital**						
L Cuneus	18	(75)	−15	−96	10	4.36
L Cuneus	18		−12	−90	18	3.91
L Middle occipital gyrus	19		−24	−95	16	3.46
R Lingual gyrus	19	(74)	27	−76	−4	4.05
R Lingual gyrus	19		33	−67	−4	3.89
R Lingual gyrus	18		15	−82	−4	3.68

All regions consist of at least 7 voxels with an uncorrected P < 0.001;

* significance P < 0.05 False Discovery Rate (FDR) correction.

**Table 4:** **Functional ROI analysis for within-group comparison of the Color group contrasting 2-relational vs. 1-relational conditions**.

**ROI**	**Hem**	**Cluster**	**BA**	**T**	**MNI coordinates**	**Contrast value**	**Uncorrected *p***
Middle frontal gyrus	L	38	6	5.59	−30, 6, 60	3.76	0.000002
Medial frontal gyrus	R	33	8	6.12	6, 18, 48	4.27	0
Middle frontal gyrus^*^	R	20	10	4.54	45, 54, −3	3.99	0.000046
Inferior occipital gyrus	R	29	18	6.02	30, −99, −3	4.49	0
Precuneus	L	12	7	5.12	−3, −66, 54	4.33	0.000006
Superior parietal lobule^*^	L	72	7	4.5	−33, −63, 54	4.53	0.000004
Superior parietal lobule^*^	R	38	7	4.58	33, −63, 57	5.09	0.000031

* Demonstrates spherical ROIs at the individual peaks of activation clusters which have been masked with the thresholded activation map for that maxima.

#### Between condition comparisons—sensory processes supporting visual properties during reasoning

First, we predicted that color and BWVC would contribute additional sensory demand during relational reasoning. This was confirmed in our behavioral results demonstrating that RTs increase as a function of complexity within BWVC and Color conditions (Table 1). By controlling for the cognitive demand of reasoning within each level of complexity we were able to assess the sensory areas that support the processing of each type of visual property (Table 2). A statistical contrast between BWVC and NC trials at 0-complexity shows correlated BOLD signal in bilateral BA 18/19. At 1-complexity level, sensory processing summates in left BA17, bilateral BA18/19, and left BA19. Finally, at 2-complexity, activation is significant in left BA18, right BA19 (bilateral lingual gyrus). Next, the statistical contrast between Color and NC show results at 1-complexity level, with sensory processing contributions from bilateral Brodmann Area 18. Color at the 2-complexity level shows sensory processing contributions from left BA18 and bilateral BA19, classically defined cortical areas of color perception.

#### Within condition comparisons of relational complexity—influence of visual properties on relational reasoning

**Figure 4:**
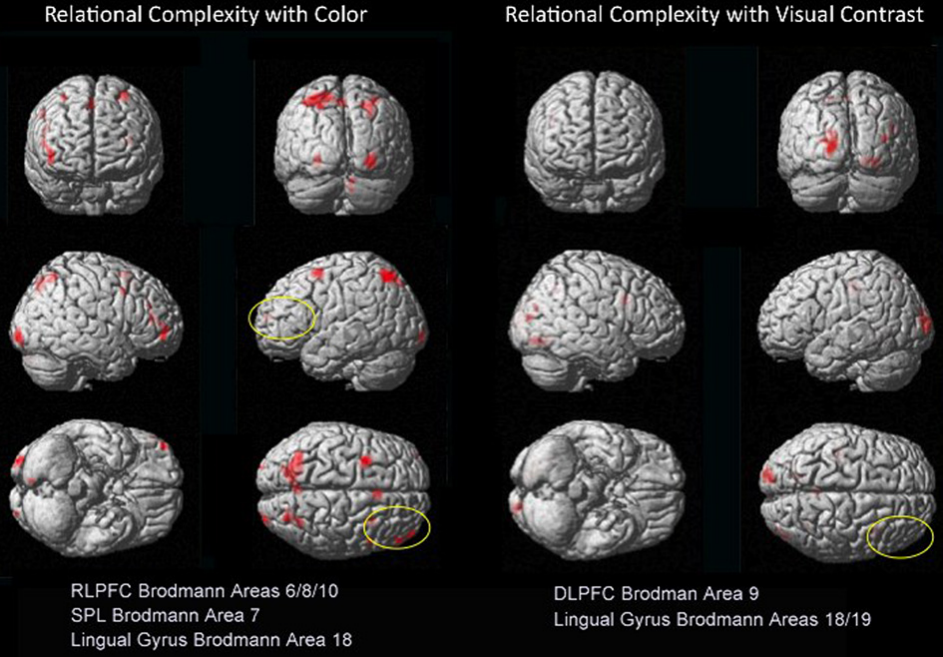
**Dissociations in prefrontal and visual-parietal regions for Color 2 > 1 and BWVC 2 > 1 relational complexity contrast results based on an RT-convolved HRF (FDR corrected, *p* < 0.05).** BOLD regions consist of at least 7 voxels (threshold *Z* > 3.09).

Within the Color 2-relational vs. 1-relational contrast, several prefrontal activations survive correction for multiple comparisons at FDR (*p* < 0.05) threshold including: right BA8, left BA6, bilateral BA10. In posterior sensory areas, significance appears in dorsal stream visual areas bilateral BA7, and BA18 in the ventral stream (Table 3 and Figure 4).

**Figure 5:**
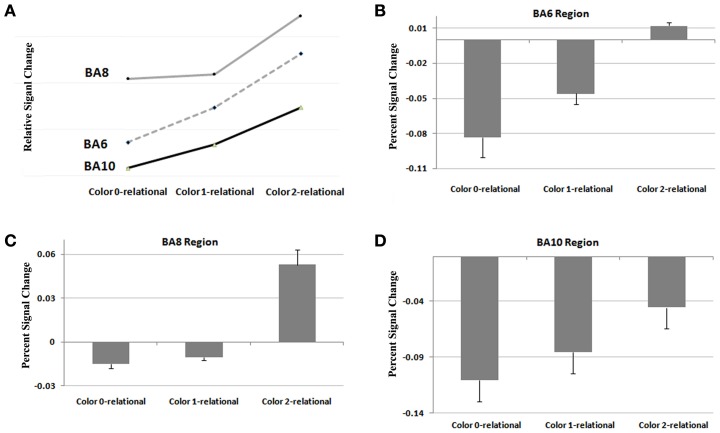
**Results from a functional ROI analysis of areas comprising the RLPFC for signal intensity at Color at 0, 1, and 2 levels of relational complexity. (A)** Relative signal change in RLPFC across levels of complexity, **(B)** Percent signal change in Brodmann 6 across levels of complexity, **(C)** Percent signal change in Brodmann 8 across levels of complexity, **(D)** Percent signal change in Brodmann 10 across levels of complexity.

To extend our examination of RC results within Color, we performed a functional and structural region of interest (ROI) analysis across different complexity levels in areas found to be significant (Tables 4, 5). A signal intensity analysis for Color 0-, 1-, and 2-relations showed that percent signal changes in BA6, 8, and 10 were significantly higher in 2-RC when compared with 0- and 1-RC (Figure 5). Of note, stimulus presentation in our task had a short inter-stimulus interval (ISI), and since RLPFC activations are substantially delayed relative to the onset of each trial (Wright et al., 2008), return toward baseline activation was observed as a negative signal change value. In this context, the trend demonstrates a parametric effect of increased activity coupled with an increase in complexity level.

**Table 5:** **Structural ROI defined based on anatomical regions identified in previous studies of relational complexity or color perception**.

**Brodmann area (BA)**	**Hem**	**Volume (cm^3^)**	***t***	**Contrast value**	**Uncorrected *p***
BA 6	L	25	1.47	0.63	0.075256
BA 8	L	9	0.11	0.06	0.450611
BA 10	L	14	1.43	0.75	0.918592
BA 7	L	14	2.78	1.33	0.004497
BA 18	L	14	1.72	0.97	0.952954

Finally, to further examine the pattern of response, Finite input response (FIR) curves were modeled for Color 1-relational and 2-relational conditions in each statistically significant ROI. The hemodynamic response modeled in FIR had similar time course trends in right middle frontal gyrus (BA10), left middle frontal gyrus (BA6), and precuneus (BA7) for each of Color 1- and 2-RC conditions. Also, FIR models in left and right superior parietal lobules (BA 7) and inferior occipital gyrus (BA18) showed a similar trend in their response pattern for Color 2-RC problems.

## Discussion

This experiment examined the influence of specific properties of the visual environment (color and BWVC) on neural systems of RC during non-verbal matrix reasoning. Our aim was to better understand how consilient real-world visual perception influences decision-making by dissociating contributions of sensory demand from higher level cognitive processing during relational reasoning. To examine this phenomenon, we used a self-paced, event-related neuroimaging design, incorporating matrix reasoning problems similar to the RPM (Raven, 1938) constructed for 0, 1, and 2 levels of RC with and without color and with BWVC.

This experiment accomplished three aims. First, we were able to dissociate the ways in which color and BWVC contribute additional sensory demand during relational reasoning. When color and BWVC are each compared with the control condition, no color line matrices, neural systems in visual cortex become more localized as problem complexity increases. Black and white visual contrast systems are more salient in the 0-relational condition. Color systems are more salient in the 1-relational condition. This is an artificial comparison because our visual world is comprised of each of these properties. In practical terms, color and visual contrast assist with our perceptual assessment of safety (demarcating boundaries), to our sense of place and capacity to navigate, and to our aesthetic sensibilities for appreciating art, design, architecture, other visual arts and aspects of our daily environments. Importantly, we demonstrate how each property contributes to sensory perception that supports sense-making during problem solving.

Second, elaborating on this discussion, color plays a specific priming role demonstrated by its influence on behavioral RT when an initial change is introduced (1-relation) such that RT significantly varies from both the control and the BWVC condition. This boost in salience is reflected as less RT needed to problem solve at this level.

**Figure 6:**
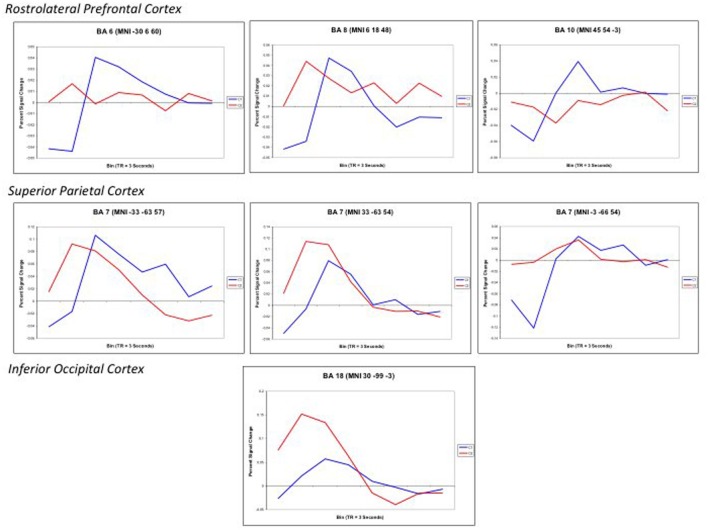
**Finite input response (FIR) curves modeling the timecourse of the RT-convolved BOLD response for Color 2-relational (red) > 1-relational contrast (blue) in each statistically significant ROI that links processing in RLPFC with the parietal and visual cortices.** ROIs are reported with the associated Brodmann Area and specific Talairach coordinates of the signal maxima from Table 3.

Lastly, as predicted, this experiment provides direct evidence for the functional connection of the RC system in the RLPFC with ventral and dorsal streams in visual cortex known to support color perception, visual-spatial processing, and navigation. This connection is demonstrated by areas of visual and parietal cortex that are activated with frontal lobe areas that specifically support relational reasoning. As described in the forthcoming paragraphs, we performed an additional region of interest analysis (ROI) to show that the time course and shape of the BOLD response in these sensory areas of the brain (visual and parietal cortices) correspond to BOLD responses assessed in the relational reasoning areas of the brain (frontal cortex) (Figures 5, 6). The BWVC condition is less important during relational reasoning. This is demonstrated on a neural level by areas of visual cortex that are activated with frontal lobe areas that support working memory, processes that are neurally and behaviorally distinct from relational reasoning (Owen et al., 1996; Ruff et al., 2003; Cowan et al., 2012).

Our fMRI results provide evidence that BWVC enlists a “sensory heuristic” during relational reasoning indicated by the multiple areas of the visual cortex listed in Table 2 that decrease in number as RC increases. Whereas color enlists a “reasoning heuristic” illustrated by the areas of cerebellum and prefrontal cortex that are activated (Rao et al., 1997; Kalbfleisch et al., 2006, 2007) as complexity increases, efficiently utilizing fewer areas of visual cortex than BWVC at higher levels of complexity. These findings demarcate how these two properties of visual perception engage different aspects of sense-making. Taken together, these results demonstrate the collective impact of multiple perceptual systems on reasoning capacities measured in such a way that plausible connections can be made between reasoning competency in the context of real life and the means by which we assess this capacity psychometrically with matrix reasoning tasks.

Consistent with previous studies (Christoff et al., 2001; Kroger et al., 2002), the no color condition in 1-relational vs. 0-relational contrast, showed no significant prefrontal voxel level activation at the uncorrected *P* < 0.001 (*Z* > 3.09) threshold. Within the no color 2-relational vs. 1-relational contrast, activations were localized in BA6, BA8, and BA10 in the RLPFC. Within the BWVC condition, the 2-relational vs.1-relational comparison, activations appear in right BA9, left postcentral gyrus, right BA40, right BA18/19 and left BA18, but are only significant at an uncorrected statistical threshold (*p* < 0.001). This validates previous studies of RC and situates this experiment to extend our knowledge of the role of RC in reasoning and problem solving.

### Influence of visual properties on sensory processing during reasoning

We confirmed our first hypothesis, that sensory properties, represented here as color and BWVC, add cognitive demand to matrix reasoning problems, resulting in a mean stepwise increase in behavioral RTs. One of the roles of the ventral visual stream is to provide visual awareness and meaningful association, “natural resources” if you will, to the dorsal visual stream to enable goal selection and action (Milner and Goodale, 2006). It helps identify context. Observing the BWVC condition at 1- and 2-levels of complexity shows the visual system becomes more efficient as complexity increases, processing visual information with inputs from a system of less-distributed areas. In contrast with this, color reasoning is only measured at statistically robust levels within 1- and 2-RC. At complexity level 1, dorsal and ventral visual areas are represented, while at complexity level 2, only the left lingual gyrus in the ventral stream is robustly active, a classic color perception area (Zeki and Marini, 1998; Gegenfurtner, 2003; Morita et al., 2004). This suggests that the extra sensory contributions from visual contrast and from color in cortex become more localized as demand increases, transferring salience to other cognitive processes. In our behavioral data, differences between these two visual properties and the control condition, no color line matrices, are statistically significant. This confirms our first hypothesis that input from the environment contributes additional sensory demand at a basic level in appropriate ways parsimonious with perceiving the visual world. On the surface this is not a novel result. However, to our knowledge, this is the first study to extend those findings into an ecologically valid model of decision-making.

### The paradoxical effect of color on behavioral performance

Unexpectedly, color had a lesser impact on RT as problem complexity increased within the color condition. One possible explanation for this paradoxical effect lies in the fact that color assists with object perception and has a role in scene segmentation and visual memory (Tanaka et al., 2001; Gegenfurtner, 2003; Werner and Chalupa, 2003; Peelen et al., 2009). Thus, color decreases cognitive demand by alerting the brain to salient properties of the visual environment. The behavioral result that RT decreased with the addition of color on the 1-relation trials (Figure 3) suggests that color facilitates associative processing by boosting salience to support cognitive performance. In fact, experiments manipulating perceptual demand have shown that response competition effects are eliminated when perceptual demand is at its highest during implicit (Rees et al., 1997; O'Connor et al., 2002; Yi et al., 2004) and explicit changes in visual awareness (Lavie, 2006). This interpretation may also be supported by emerging evidence that general intelligence (IQ) can be predicted by sensory integration and how well-someone suppresses interference from visual motion (Melnick et al., 2013).

The priming effect of color on salience addresses the question of how neural systems of RC converge with sensory visual properties to support reasoning and problem solving. When each property is examined within RC (2-relation vs. 1-relation), we demonstrate a double-dissociation between the areas of the prefrontal and visual-parietal cortices for color and BWVC (Table 3, Figure 4). During color RC, processing from RLPFC works in concert with areas from dorsal and ventral visual streams. The left lingual gyrus in the ventral stream is a classic color perception area. In addition, activity from parietal areas in BA7 (cuneus and superior parietal lobule) in the dorsal visual stream that support RC performance during color conditions is accounted for theoretically and experimentally as the ventral stream perceives objects and the dorsal stream orients to location (Ungerleider et al., 1998).

The neural correlates that accompany increases in RT suggest that color is a visual property that contributes to cognitive demand in RC. The between condition contrast, color vs. no color, illustrated that the addition of color to RC trials requires additional processing from parietal and ventral occipital areas to process visual information. This is important in light of the evidence that perception of context is engaged early in decision-making and relies on the dorsal stream as a primary pathway for information transfer between the visual and frontal cortices (Kveraga et al., 2007, 2011). As a control condition for color, the BWVC condition demonstrates that color has an additional effect on cognitive processing beyond the presence of a degree of change between no color line matrices and those with BWVC.

### The impact of color on relational complexity

An additional question motivating the experiment was how the actual measure of RC (contrasting 2- to 1-relational performance) would be influenced by color? We assessed this by examining the Color 2 vs. Color 1 within relational contrast. Our results provide evidence of the recruitment of systems beyond those in the prefrontal cortex that appear necessary for color relational reasoning, extending the known system in RLPFC to include relevant superior parietal and inferior occipital areas (Table 3 and Figure 4). The neural systems supporting color RC are comprised of functional areas underlying different components of our task, including a suite of areas in middle frontal gyrus (left BA6, right BA8, and bilateral BA10 biased toward the right hemisphere), right BA18, and bilateral BA7. Coordinates of the left middle frontal gyrus in our experiment are almost identical to the ones reported by Christoff et al. (2001), though we report a bilateral signal in BA10 biased in strength to the right hemisphere. Bunge et al. (2009) report findings to illustrate the differential roles of the left and right hemispheres of the RLPFC. Specifically, the left hemisphere is the primary source of the integration of relations, while the right hemisphere attends to the set maintenance of the complexity. While we report bilateral activation of RLPFC during color RC, only the right hemisphere survives statistical correction. Collectively, the involvement of left BA6 in spatial working memory (Glahn et al., 2002; Wager and Smith, 2003; Tanaka et al., 2005), the role of BA8 in managing uncertainty and approximation (Volz et al., 2005), and BA10′s role in RC (Christoff et al., 2001; Kroger et al., 2002), support the suggestion that these areas appear to function in a set capacity, to help the brain maintain salience with overall context and state while more task specific processes occur in the opposite hemisphere. Undoubtedly, higher cortical processes interact with perceptual neurological systems and share their cognitive resources (Petrides, 2005; Simmons et al., 2007; Mahon and Caramazza, 2009; Whitney, 2009). It has been suggested that the occipito-parietal lobe is sensitive to the complexity of relational information (Phillips and Niki, 2006; Wendelken et al., 2008b), and that parietal neurons support associative representation and “contribute to a distributed network that supports learned associations during visual stimulation and working memory periods” (Fitzgerald et al., 2011). These areas collectively demonstrated in our results suggests a parsimonious outcome for how RC merges with other processes to make visual reasoning possible in everyday life.

Color 2-RC problems in our task recruited bilateral BA7. Activations in BA7 have been previously associated with working memory, setting attention priority, potential movement coding (Shibata and Ioannides, 2001; Molenberghs et al., 2007) and visual-spatial imagery processes (Ruff et al., 2003). The fit curves from our ROI analysis demonstrate that the engagement of the superior parietal lobule (BA7) was modulated by task complexity and also paralleled the trend of the hemodynamic response observed in the middle frontal areas (Figure 6). This correlate suggests that parietal engagement in color RC supports activity in these prefrontal areas during RC. In addition to its role in spatial shifting, Wendelken et al. (2008a) submit that BA7 is sensitive to relational structure as well as grouping and serial order combinations. This is particularly important because they make their case for the role of BA7 as a flexible and general support structure for spatial information.

Our color RC task engages aspects of two systems that have been historically distinct in their roles, ventral and dorsal visual pathways (Mishkin et al., 1983; Kravitz et al., 2011) and parietal pathways designated for perception and action (Milner and Goodale, 2006). The color RC system captured here points to the type of top–down influence from higher-order brain areas that guide goal-directed behavior (Sakai, 2008) and facilitate object recognition when compared to achromatic stimuli (Kveraga et al., 2007). These areas work in concert as the posterior parietal cortex has limited memory capacity on its own to retain rich representations of the visual world (Todd and Marios, 2004).

The summative functional system represented in this data suggests there is a boundary condition delineating color's role in priming (Zago et al., 2005) and dissociates salience at two different levels of visual complexity. On a behavioral level, this enhances the visual target over background objects. The supportive effect of color at the highest level of RC suggests there is a boundary condition present in how the brain utilizes or encodes color perception during higher-level cognition. As our behavioral data indicate and the robust bilateral activations present in the cuneus and lingual gyrus present when color is introduced at 1-RC (Table 2) vs. 2-RC, activity in the visual system has pared down to the left lingual gyrus in the absence of competing visual complexity. In this case, it may be perceived as its own object *per se*, imposing cognitive demand that results in a longer RT. The practical outcome of this result provides a general principle for using color to prime or ready the cortex for more complex problem solving, a variable easily manipulated in the design of virtual worlds and gaming environments.

## Conclusion

Our data point to two plausible mechanisms that can be probed in future experiments. First, prior evidence suggests that color engages perceptual processes much faster than was previously thought (Holmes et al., 2008; Seymour et al., 2009) and takes longer to bind (Bartels and Zeki, 2006), lending an explanation for how it imposes influence on contextual salience. Second, it has also been suggested that activity in V4, the “color” area in visual cortex, extends beyond sensory memory (which diminishes in less than 500 ms) and is a fundamental support for short-term memory bridging between conscious and non-conscious processing (Sligte et al., 2009). Taken together, these results provide a plausible explanation for the means by which color influences sense-making from a constructivist perspective.

Prior to this, the effect of color on the mental representations of relations and the level of visual complexity of a task was unknown. When color is “added” to the environment of our task conditions, perceptual systems in the brain are enlisted to detect it. Further, the facilitation effect we find illustrated behaviorally at 1-relation, shows that when color is present in a moment of reasoning, it is incorporated into and enhances the mental representation of the stimulus (i.e., its navigational significance). This is an important distinction, the distinction between perception of color as a general property in the visual environment and color as an object property of a stimulus in the neuroimaging environment. Early experiments exploring aspects of complexity within several different visual properties determined that features of symmetry and context reduce the psychological perception of complexity (Chipman, 1977), a potential explanation for color's influence on behavior during 1-relational problem solving.

It is worth noting a potential limitation but important feature of our study. We did not vary color as a dimension of RC *per se*. Our choice to use the color blue as a starting point came from the fact that it is a color most people can perceive, including those with color-blindness (Deeb, 2004). Had we introduced other colors into the experiment to vary color as a specific aspect of RC, we would have introduced an attention confound as some colors, such as red and green, are known from an evolutionary standpoint to have greater salience (Gerl and Morris, 2008). Also, blue/yellow color vision phylogenetically preceded red/green color vision and is perceived with black/white (rods) to improve scene and context detection. Introducing red/green color heuristics into the experiment could have confounded our ability to measure the effects of color complexity on rational scene analysis and problem solving. No activations were measured in the frontal cortex in the contrasts Color 1 vs. NC 1 or Color 2 vs. NC 2-RC. This result aligns with a previous report that the frontal cortex is not engaged in selecting color on its own (Rowe et al., 2005).

In total, this experiment yields results that discern rules for how the brain employs different and specific reasoning and sensory heuristics for processing color and visual contrast to show how these features are assimilated during problem solving. By demonstrating this within the context of matrix reasoning, we have preserved the relevance of neuroimaging to inform our understanding of the assessment of human intelligence and to influence the design and optimization of formal and informal physical and virtual learning environments.

### Conflict of interest statement

The authors declare that the research was conducted in the absence of any commercial or financial relationships that could be construed as a potential conflict of interest.
